# Duplication of the prothrombin gene is associated with a significant increase in thrombin generation

**DOI:** 10.1016/j.rpth.2026.103386

**Published:** 2026-02-02

**Authors:** Annelie Siegemund, Thomas Siegemund, Hagen Bönigk, Kristina Schlosser, Katja Konn, Sabine Keil, Sirak Petros

**Affiliations:** 1 Medical Service Center Limbach Magdeburg, Center of Blood Coagulation Disorders and Vascular Diseases, Magdeburg, Germany; 2 Medical Service Center Laboratory Dr Reising-Ackermann & Colleagues – Leipzig, Department of Molecular Genetics, Leipzig, Germany; 3 Medical Service Center Laboratory Dr Reising-Ackermann & Colleagues – Zwickau, Cytogenetic Laboratory, Zwickau, Germany; 4 Medical Intensive Care Unit, University of Leipzig, Leipzig, Germany

**Keywords:** prothrombin activity, prothrombin gene duplication, prothrombin mutation, thrombin generation, venous thromboembolism

## Abstract

**Background:**

Prothrombin gene mutations can be associated with either a thrombotic or a bleeding risk. Genomic studies and coagulation workup can provide valuable information to better understand their clinical importance.

**Key Clinical Question:**

We describe the case of a woman with a duplication of the entire prothrombin gene.

**Clinical Approach:**

A 42-year-old woman presented for thrombophilia screening following a history of unprovoked arterial and superficial venous thrombotic episodes. Coagulation workup demonstrated a marked increase in prothrombin levels and *ex vivo* thrombin generation. Genetic analysis revealed a duplication of at least 307.9 kb (maximum 366.7 kb): arr[ChRCh38]:11p11.2(46,455,533-46,763,446)x3, encompassing the entire prothrombin gene and 6 adjacent protein-coding genes (HARBI1, ATG13, ARHGAP1, and ZNF408 completely involved, and AMBRA1 and CKAP5 partially involved).

**Conclusion:**

The present case demonstrated duplication of the entire prothrombin gene, associated with a significant hypercoagulable risk, a finding not previously reported in the literature.

## Essentials

• A prothrombin gene duplication was detected in a woman with a history of unprovoked thromboses.
• Gene analysis revealed a duplication of at least 307.9 kb (maximum 366.7 kb).
• This duplication encompassed the entire prothrombin gene and 6 adjacent protein-coding genes.
• The mutation was associated with markedly increased prothrombin levels and thrombin generation.

## 1 Introduction

The well-known prothrombin (F2) gene sequence variant, NM_000506.3:c.∗97G>A (commonly known as G20210A), results in a markedly elevated prothrombin concentration that is associated with an increased risk of venous thromboembolism [1]. Here, we present the case of a 42-year-old woman with a duplication of the entire prothrombin gene, which has not yet been reported in the literature in the context of increased prothrombin levels and *ex vivo* thrombin generation (TG).

## 2 Case Presentation

The woman suffered an unprovoked arterial thrombotic occlusion of the left femoral artery 10 years ago. She was started on combined secondary prophylaxis with aspirin and the vitamin K antagonist phenprocoumon. The latter was stopped after a year, while aspirin was continued as long-term maintenance treatment. Subsequently, the patient was diagnosed with repeated episodes of spontaneous thrombophlebitis with superficial venous thrombosis without a provoking event. She was treated for the latest episode with subcutaneous fondaparinux, which was discontinued by her family physician 2 weeks prior to presentation for thrombophilia screening at our center. On presentation, the woman was not pregnant. Her body mass index was 35.3 kg/m^2^. She did not use hormonal contraception. She did not have any comorbidities. She has neither undergone a surgical procedure nor suffered any trauma. Her parents were already deceased. She reported that her father suffered an unprovoked deep vein thrombosis and pulmonary embolism.

The patient’s coagulation results are presented in Table 1; the prothrombin level was markedly elevated. Standard thrombophilia screening, including genetic testing for the factor V Leiden mutation, was negative.

**Table 1:** Laboratory data of the patient on presentation

Variable	Concentration	Reference range
INR	0.97	<1.2
PTT (s)	24.6	23-31.9
Fibrinogen (Clauss method, g/L)	3.86	1.8-3.5
Thrombin time (s)	17.7	<20
D-dimer (mg/L)	0.64	<0.5
Prothrombin fragments F1 + 2 (pmol/L)	296.8	63-307
Prothrombin (IU/mL)	>1.80	0.70-1.20
Protein C (IU/mL)	1.08	0.70-1.40
Protein S (IU/mL)	1.03	0.65-1.39
Antithrombin (IU/mL)	1.09	0.83-1.15
Lupus anticoagulant	Negative	Negative

INR, international normalized ratio; IU, International Units; PTT, partial thromboplastin time.

TG measurements were performed with the ST Genesia (Stago) automated analyzer using the STG-ThromboScreen assay (Stago) as described earlier [2]. In short, this system measures TG as in the calibrated automated thrombogram, with 80 μL of platelet-poor plasma mixed with 20 μL of trigger solution. The samples were preheated at 37 °C. Coagulation was then initiated by the addition of 20 μL of buffer containing a fluorescent substrate (Z-Gly-Gly- Arg-AMC) and CaCl_2_. To correct for differences in plasma color, each plasma measurement was calibrated against the same plasma mixed with 20 μL of thrombin calibrator. The fluorescence of 7-amino-4-methylcoumarin was measured using a Fluoroskan Ascent fluorometer (Thermo Fischer Scientific). In contrast to the calibrated automated thrombogram, a single overall calibration, representing the activity of a known thrombin concentration, is performed once daily. Fluorescence was measured using a 377/440 nm filter set (excitation/emission). TG was performed in duplicate. Each reagent kit includes a reference plasma for normalizing TG values. The following parameters were measured: lag time, peak height, time to peak, endogenous thrombin potential, velocity index, and start tail. Normalized parameter results were automatically calculated by the system using the following formula: patient sample result/reference plasma result × the activity assigned to the particular lot and parameter of the reference plasma. The normalized results are expressed as percentages or ratios. Data from this patient, from a woman of the same age with a homozygous prothrombin G20210A mutation, and from the reference plasma are presented in the Figure. Data for the TG variables are presented in Table 2 as absolute and normalized values relative to the reference plasma.

**Figure:**
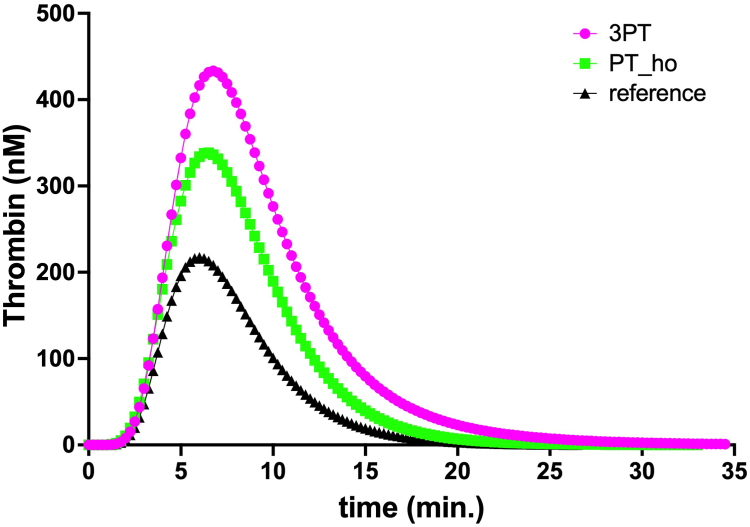
Thrombin concentration assessed with the STAGO STG-ThromboScreen assay for the presented case (3PT), a patient with a homozygous G20210A mutation (PT_ho), and from reference plasma (reference).

**Table 2:** Thrombin generation parameters for the index patient and a patient of the same age with a homozygous prothrombin G20210A mutation

Variable	Index patient		Patient with PT-ho	
	Absolute values	Normalized values	Absolute values	Normalized values
Lag time (min)	3.06	1.42	2.83	1.31
Peak height (nM)	433.4	177.6%	338.5	136.0%
Time to peak (min)	6.75	1.45	6.45	1.41
ETP (nM.min)	3456	241.7%	2451	161.1%
Velocity index (nM/min)	148.3	128.1%	117.1	92.69%
Start tail (min)	24.19	1.35	21.01	1.11

Normalized values are given either as a ratio or a percentage of those from reference plasma generated during the individual test runs.

ETP, endogenous thrombin potential; PT-ho, homozygous prothrombin G21010A mutation.

Molecular genetic analysis was performed using a next-generation sequencing panel on DNA extracted from the patient’s EDTA blood. The coding regions, as well as the proximal and distal intron regions of the *F2* gene, were enriched by probe hybridization (Library Preparation EF 2.0 with Enzymatic Fragmentation and Twist Universal Adapter System, TWIST Bioscience) and subsequently sequenced using MiSeq (Illumina). Varvis software (Limbus Medical Technologies) and Sequence Pilot software (JSI Medical Systems) were used to align reads to the human reference genome GRCh37/hg19 (University of California Santa Cruz Genomics Institute) and to conduct copy number variation analysis.

Next-generation sequencing-based copy number variation analysis revealed a duplication of the entire *F2* gene. No clinically relevant single-nucleotide variations were detected in the *F2* gene of the patient. To confirm the duplication by an independent approach, array-comparative genomic hybridization was performed using the Oxford Gene Technology (4 × 180 k) Constitutional v3 platform (Oxford Gene Technology), with a gender-matched normal human DNA pool (Sysmex) as the control and the human genome assembly GRCh38/hg38 as the reference. An InnoScan 710 Microarray Scanner and Mapix v.9.1.0 (Innopsys) were used to detect and analyze fluorescence levels. Results were interpreted using Cytosure Interpret Software v4.11.58 (Oxford Gene Technology). The array-comparative genomic hybridization showed a duplication of at least 307.9 kb (maximum 366.7 kb): arr[ChRCh38]:11p11.2(46,455,533-46,763,446)x3, encompassing the entire *F2* gene and 6 adjacent protein-coding genes (HARBI1, ATG13, ARHGAP1, and ZNF408 completely involved, and AMBRA1 and CKAP5 partially involved). No definite dose sensitivity is known for any of these genes. Only for the partially affected genes, some predictors indicate possible haploinsufficiency and triplosensitivity, respectively.

Both parents of our patient were already deceased, and other first-grade family members were not available for genetic and coagulation analysis.

## 3 Discussion

To the best of our knowledge, this is the first reported case of duplication of the entire *F2* gene, with a marked increase in prothrombin levels and TG. A significantly smaller duplication (38 kb) was reported in the ClinVar database (National Library of Medicine) as a variant of unknown significance, including the *F2* gene and the adjacent *ZNF408* gene. The reported condition in ClinVar was associated with a congenital prothrombin deficiency, but detailed clinical information was not provided (accession number: VCV003244538.1). Additionally, 2 slightly larger duplications were reported in the DatabasE of genomiC varIation and Phenotype in Humans using Ensembl Resources [DECIPHER] database (European Molecular Biology Laboratory - European Bioinformatics Institute), covering the same genes as in our patient (patient 288560; 336.17 kb and patient 307922; 366.59 kb). In these cases, the duplications were assigned as likely benign and uncertain, respectively. However, the coagulation phenotypes were not reported.

A further investigation of family members would have been ideal. However, both parents of the woman were already deceased, and other first-grade family members were not available.

Gehring et al. [3] showed in an *in vitro* study that the prothrombin G20210A mutation is a gain-of-function variant, leading to increased mRNA accumulation and protein synthesis. Recently, Wen et al. [4] reported a novel heterozygous prothrombin mutation, p.Ile441Met (c.1323A>G), in a Han Chinese family with a history of venous thromboembolism [4]. In that cohort, TG was higher than in the control sample, whereas prothrombin activity was markedly lower than in our patient. The extent of TG in the presented case was even more pronounced than that of a patient with a homozygous prothrombin G20210A mutation. Therefore, we speculate that duplication of the entire *F2* gene might also lead to increased prothrombin synthesis.

In our patient, the level of prothrombin fragments 1 + 2 was within the normal range. Therefore, the pathomechanism leading to thrombosis related to this mutation is probably associated with the higher amounts of thrombin once TG is triggered, rather than with an ongoing TG *in vivo* [5].

In conclusion, the detected prothrombin gene duplication in our patient may have been associated with markedly increased prothrombin levels and *ex vivo* TG.
